# Optimization and Reproducibility of Aortic Valve 18F-Fluoride Positron Emission Tomography in Patients With Aortic Stenosis

**DOI:** 10.1161/CIRCIMAGING.116.005131

**Published:** 2016-10-18

**Authors:** Tania A. Pawade, Timothy R.G. Cartlidge, William S.A. Jenkins, Philip D. Adamson, Phillip Robson, Christophe Lucatelli, Edwin J.R. Van Beek, Bernard Prendergast, Alan R. Denison, Laura Forsyth, James H.F. Rudd, Zahi A. Fayad, Alison Fletcher, Sharon Tuck, David E. Newby, Marc R. Dweck

**Affiliations:** From the BHF/Centre for Cardiovascular Science (T.A.P., T.R.G.C., W.S.A.J., P.D.A., D.E.N., M.R.D.), Clinical Research Imaging Centre, Queen’s Medical Research Institute (C.L., E.J.R.V.B., A.F.), and Edinburgh Clinical Trials Unit, Western General Hospital (L.F.), University of Edinburgh, United Kingdom; Translational and Molecular Imaging Institute, Icahn School of Medicine at Mount Sinai, New York (P.R., Z.A.F.); Guy’s and St Thomas’ Hospitals NHS Foundation Trust, London, United Kingdom (B.P.); Institute for Education in Medical and Dental Sciences, University of Aberdeen, United Kingdom (A.R.D.); Division of Cardiovascular Medicine, University of Cambridge, United Kingdom (J.H.F.R.); and Wellcome Trust Clinical Research Facility, Western General Hospital Edinburgh, United Kingdom (S.T.).

**Keywords:** 18F-Fluoride, aortic valve stenosis, calcification, disease progression, echocardiography, positron emission tomography

## Abstract

**Background—:**

18F-Fluoride positron emission tomography (PET) and computed tomography (CT) can measure disease activity and progression in aortic stenosis. Our objectives were to optimize the methodology, analysis, and scan–rescan reproducibility of aortic valve 18F-fluoride PET-CT imaging.

**Methods and Results—:**

Fifteen patients with aortic stenosis underwent repeated 18F-fluoride PET-CT. We compared nongated PET and noncontrast CT, with a modified approach that incorporated contrast CT and ECG-gated PET. We explored a range of image analysis techniques, including estimation of blood-pool activity at differing vascular sites and a most diseased segment approach. Contrast-enhanced ECG-gated PET-CT permitted localization of 18F-fluoride uptake to individual valve leaflets. Uptake was most commonly observed at sites of maximal mechanical stress: the leaflet tips and the commissures. Scan–rescan reproducibility was markedly improved using enhanced analysis techniques leading to a reduction in percentage error from ±63% to ±10% (tissue to background ratio MDS mean of 1.55, bias −0.05, limits of agreement −0·20 to +0·11).

**Conclusions—:**

Optimized 18F-fluoride PET-CT allows reproducible localization of calcification activity to different regions of the aortic valve leaflet and commonly to areas of increased mechanical stress. This technique holds major promise in improving our understanding of the pathophysiology of aortic stenosis and as a biomarker end point in clinical trials of novel therapies.

**Clinical Trial Registration—:**

URL: http://www.clinicaltrials.gov. Unique identifier: NCT02132026.

## Abstract 2

Supplemental Digital Content is available in the text.

Aortic stenosis is the most common form of valve disease in the Western world and a major healthcare burden that is set to treble by 2050. However, we currently lack any disease-modifying therapies. Calcification seems to be the predominant pathological process driving disease progression, leading to major interest in novel treatment strategies aimed at reducing calcification activity in the valve.^1^ However, assessing the efficacy of new therapies requires large trials with prolonged follow-up to demonstrate an impact on disease progression and clinical end points.^2^ A noninvasive imaging technique capable of measuring calcification activity in the valve would be highly desirable to assess treatment efficacy in phase 2 clinical trials.

**See Editorial by Chang and Chareonthaitawee**

**See Clinical Perspective**

18F-Fluoride is a positron-emitting radiotracer that binds to regions of newly developing microcalcification beyond the resolution of computed tomography.^3^ It is readily taken up by the valves of patients with aortic stenosis, and, on histology, correlates with markers of calcification activity.^4^ Importantly, this technique predicts disease progression both with respect to echocardiography and computed tomography (CT) calcium scoring and with respect to adverse cardiovascular events.^5–7^ 18F-Fluoride positron emission tomography (PET) imaging therefore holds major promise as a marker of calcification activity in aortic stenosis and is an exploratory secondary end point in the ongoing SALTIRE2 clinical trial (NCT02132026). Briefly, this is a randomized controlled trial investigating the ability of therapies targeting calcium metabolism (denosumab and alendronic acid) to modify disease progression in aortic stenosis.

Here, we sought to optimize 18F-fluoride PET scanning of the aortic valve, reduce the effects of cardiac motion, and assess the scan–rescan reproducibility of this technique to inform its future application as a novel biomarker of calcification activity in clinical trials.

## Methods

### Study Population

Patients aged >50 years with mild, moderate, and severe calcific aortic stenosis were recruited prospectively from outpatient clinics at the Edinburgh Heart Center. Aortic stenosis severity was determined by clinical echocardiograms and graded according to according to American Heart Association/American College of Cardiology guidelines. This is a substudy of the ongoing SALTIRE2 clinical trial (NCT02132026), and consequently patients had to meet the same exclusion criteria as those entering the main trial. These included renal failure and women of childbearing potential (full list in Table I in the Data Supplement). The study was approved by the Scottish Research Ethics Committee and has a Clinical Trial Authorization from the Medicines and Healthcare products Regulatory Authority of the United Kingdom. It was performed in accordance with the Declaration of Helsinki. All patients gave written informed consent.

### Initial Image Acquisition and Analysis

Each patient underwent 18F-fluoride PET and CT scanning on 2 occasions. Patients were given 25 mg of oral metoprolol if their resting heart rate was >65 beats/min before being administered 125 MBq of 18F-fluoride IV. After 60 minutes, patients were imaged with a hybrid PET and CT scanner (Biograph mCT; Siemens). Attenuation-correction CT scans were performed before acquisition of PET data in list mode using a single 30-minute bed position centered on the valve in 3-dimensional mode. Finally, ECG-gated aortic valve CT calcium scoring and contrast-enhanced CT angiography were performed in diastole and in held expiration.

CT calcium scoring was performed by an experienced operator using dedicated software (Vitrea Advanced; Toshiba Systems) on axial views, with care taken to exclude calcium originating from the ascending aorta, left ventricular outflow tract, and coronary arteries. The calcium score was recorded in Agatston units.

Analysis was performed using an OsiriX workstation (OsiriX version 3.5.1 64-bit; OsiriX Imaging Software, Geneva, Switzerland). As previously reported, regions of interest were drawn around the perimeter of the valve on the fused nongated PET and noncontrast CT images.^6^ These generated mean and maximum standard uptake values (SUV) for each slice. Averaging these values across the entire valve produced whole valve SUV_mean_ and SUV_max_ values, respectively. These SUV values were then corrected for blood-pool activity to generate tissue to background ratio (TBR): whole valve TBR_mean_ and TBR_max_. The blood-pool uptake was determined using SUV_mean_ values averaged from across regions of interest drawn on 5 contiguous slices in the brachiocephalic vein. For consistency, the most caudal region of interest was positioned at the point where the innominate vein joined the brachiocephalic vein.^6^

To optimize the spatial localization and scan–rescan reproducibility of 18F-fluoride PET-CT imaging, we assessed different approaches to both image acquisition and image analysis.

### Optimization of PET Image Acquisition

#### Contrast CT of Aortic Valve

**Figure 1.**
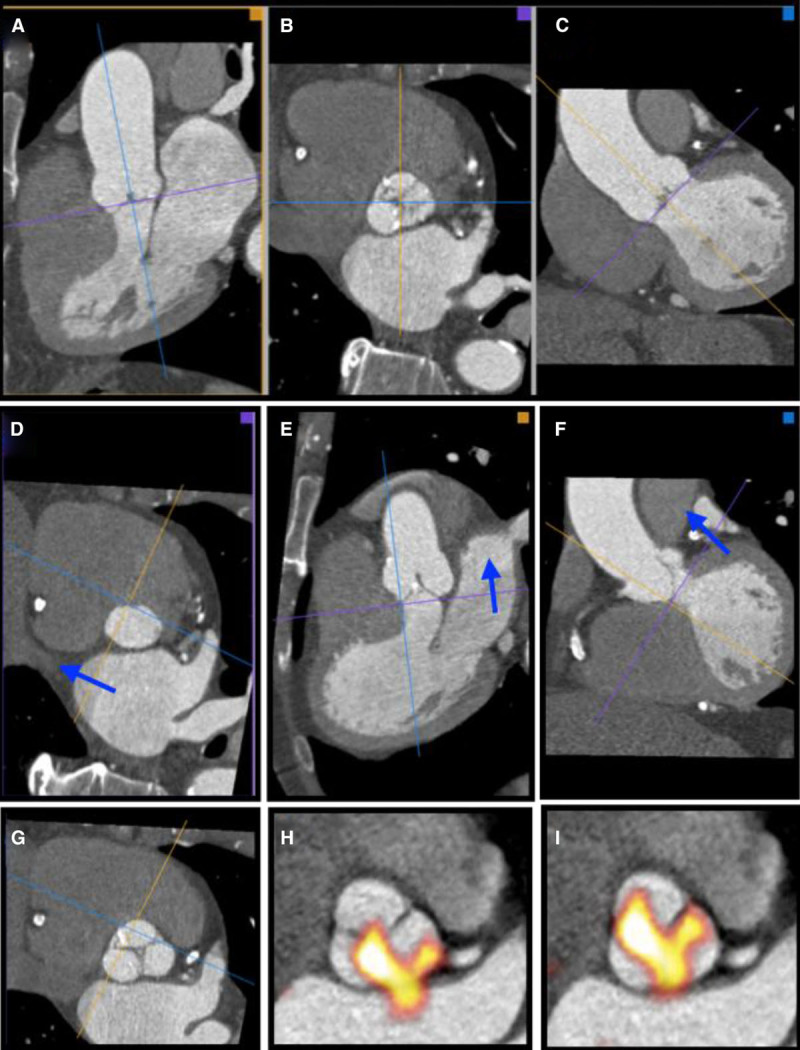
Creation of coregistered en face short-axis positron emission tomography (PET)/computed tomography (CT) images of the aortic valve. First, the CT angiogram is reorientated to get into the approximate plane of the aortic valve by lining up the axial cross hair (purple in this example) using the images in the coronal (**A**) and sagittal planes (**C**). This creates an approximate cross-sectional image of the aortic valve in the axial frame (**B**). Scrolling down in the axial frame, the center of the crosshairs is then placed over the exact point at which the right coronary cusp disappears, identifying the base of that leaflet (**D**). Similarly, the base of the noncoronary cusp is identified, and orthogonal planes adjusted so that the purple plane goes through the base of both these 2 cusps (**D**). Finally, the base of the left coronary cusp is found by rotation of the axial crosshairs so that first the cusp comes into view. The image is then slowly rotated in the opposite direction until the point where the leaflet first disappears (the base) is again found (**F**). This produces an en face image of the valve aligned with the base of all 3 leaflets (**G**). Adjacent 3-mm slices are then created in that plane and used for subsequent assessment. These slices are fused with the 18F-fluoride PET images (**H**) and careful coregistration performed in 3 dimensions to ensure accurate alignment between the PET and CT images (**I**).

Our original technique required the reorientation and coregistration of noncontrast CT images of the aortic valve. This technique posed several challenges, particularly with respect to aligning with the true plane of the valve and accurately defining its perimeter. Moreover, the structure of individual leaflets was not visible on these scans precluding more detailed localization of 18F-fluoride uptake. Contrast CT offered potential solutions to these challenges given its superior anatomic detail and the well-established methodology for finding the true plane of the valve^8^ (Figure 1).

#### ECG-Gated PET Data

PET is susceptible to motion, limiting accurate coregistration and the spatial assessment of PET activity within the valve. As a solution, we used ECG gating of list-mode PET data. These data were reconstructed into 4 gates at 25% intervals of the cardiac cycle. Only data acquired between 50% and 75% of the RR interval were assessed because this period corresponds with diastole when cardiac motion is at a minimum. Given that 3 quarters of the PET data are therefore discarded, the bedtime was increased to 30 minutes to preserve signal to noise ratio.

### Optimization of PET Image Analysis

#### Measurement of Blood-Pool Activity

The stability of blood-pool measurements in the SVC for 18F-fluoride–based tracers has recently been questioned,^9^ and we were concerned about variation in the measured blood-pool activity at different levels of the brachiocephalic vein. We reasoned that this may be explained by the relatively small diameter of this vein rendering it susceptible to partial volume effects, amplified by the low PET signal in surrounding lung tissue (especially in the cranial aspects of the brachocephalic vein). We hypothesized that sampling blood-pool activity from the center of the right atrium (a much larger structure) may improve the ease and accuracy with which these measurements could be made and the consequent scan–rescan reproducibility. Using the same coregistered PET and CT images of the heart, reorientated to the plane of the valve, a 2-cm^2^ region of interest was drawn in the center of the right atrium at the level of the right coronary ostium and again in the same position one slice superiorly. Averaging the mean SUV for these 2 slices gave an alternative measure of blood-pool activity, which was used to correct valvular uptake measurements using 2 different approaches. First, we used the conventional method of dividing aortic valve SUV measurements by the blood pool to generate TBR values. Second, we subtracted the blood-pool value from the valvular uptake, to generate the corrected aortic valve SUV as described recently.^9^

#### Most Diseased Segment and Whole Valve Approach

One of the biggest difficulties in quantifying uptake in the valve is defining its limits in the z-plane. To overcome this challenge, our original whole valve technique was compared with a most diseased segment (MDS) approach where the 2 contiguous slices with the highest SUV values (frequently in the center of the valve) were averaged to generate SUV_MDSmean_, SUV_MDSmax_, and corresponding TBR values. This is similar to the approach previously used for quantifying 18F-fluorodeoxyglucose uptake in carotid and aortic atheroma.^10^

### Scan–Rescan Reproducibility

Scan–rescan repeatability and intra- and interobserver reproducibility of valvular 18F-fluoride PET quantification was assessed for each of the established and novel image analysis approaches described above. Two experienced operators (T.P. and T.C.) quantified uptake values on each of the scan pairs, on 2 occasions separated by ≥2-week interval to avoid recall bias. Observers were blinded to both their own previous measurements and those of the other operator.

### Spatial Resolution

The effect of our modifications on spatial resolution and scan–rescan reproducibility were then assessed in comparison with the original approach. First, we assessed the ability of the technique to localize increased 18F-fluoride activity to individual valve leaflets and their different regions. This was done visually using a standardized method for windowing the fused PET/CT images that incorporated the blood-pool activity in right atrium as the minimum. Scan–rescan and observer agreements were assessed.

### Statistical Analysis

Continuous variables were expressed as mean±SD, and categorical variables were expressed as total and percentage. Kappa statistics (with 95% confidence intervals) were used to measure the intraobserver and scan–rescan agreement in presence or absence of 18F-fluoride uptake across coronary cusps. The κ values were interpreted as follows: poor <0.20, fair 0.21 to 0.4, moderate 0.41 to 0.60, good 0.61 to 0.80, and very good >0.81.

Intraobserver, interobserver, and scan–rescan reproducibility of several 18F-fluoride PET uptake approaches were analyzed and presented using Bland–Altman analysis and percentage error.^11^ Variability in the different techniques was expressed using the width of the 95% limits of agreement from Bland–Altman analyses. For the final approach, we considered the scan–rescan reproducibility to be good and acceptable for use in our future trial if the width of the 95% limits of agreement were within ±0.2 for the TBR_MDSmean_ measurements. Percentage errors for the mean bias were calculated using twice the SD of the difference divided by the overall mean measurements. The intraclass correlation coefficient was used to examine the reliability for both intra and interobserver variability.

Statistical analysis was performed using SAS for Windows version 9.4. Graphs were produced using PRISM version 6.0 for Mac.

## Results

### Patient Characteristics

Fifteen patients (73±7 years, 67% men) had 2 scans (Table 1), 3.9±3.3 weeks apart between November 2014 and May 2015. Seven patients had mild aortic stenosis, 4 had moderate and 4 had severe aortic stenosis. In 3 participants, the between-scan interval exceeded 4 weeks (5 weeks, 8 weeks, and 14 weeks). The dose of 18F-fluoride was similar on each visit (123±8 and 125±4 MBq; *P*=0·49).

**Table 1.**
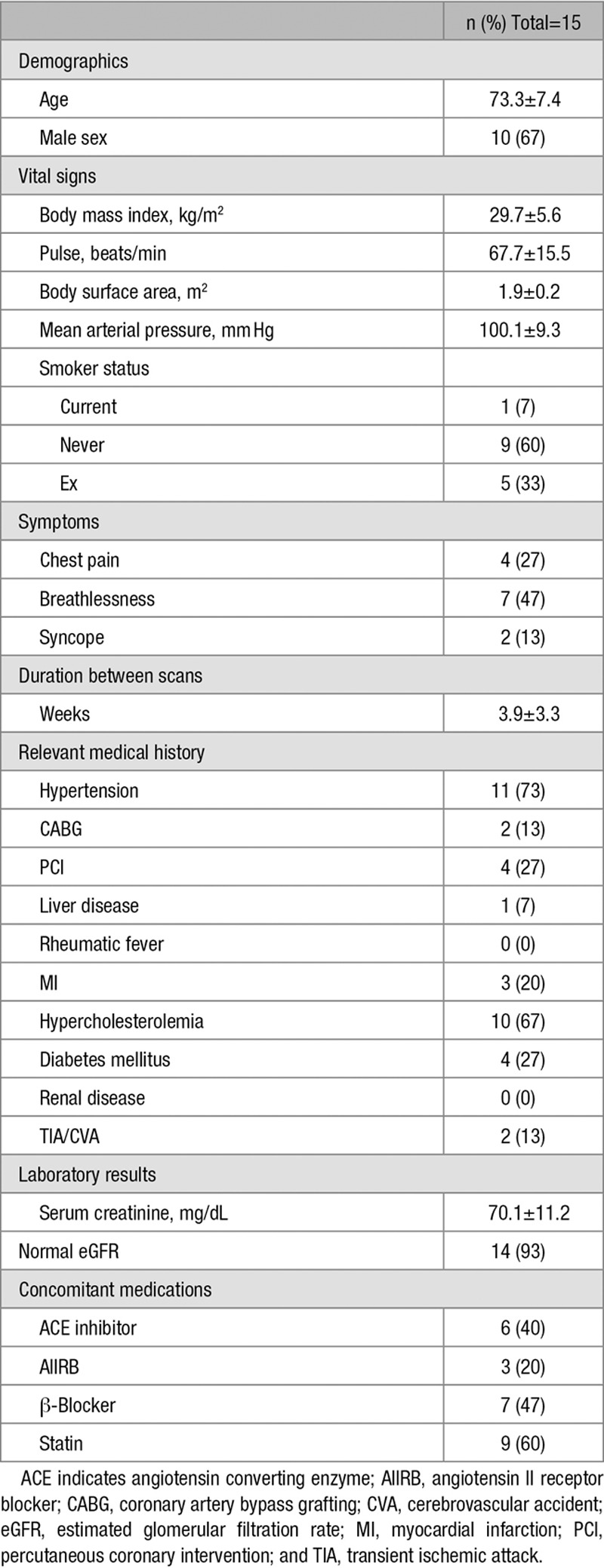
Patient Characteristics

### Altered PET Acquisition and Image Quality

Good image quality allowing complete image analysis was achieved on all 15 scan pairs. The prolonged bedtimes of 30 minutes did not result in increased patient motion during the 18F-fluoride PET scans. The impact of each stepwise change in the acquisition and analysis protocol on scan–rescan reproducibility is summarized in Table 2.

**Table 2.**
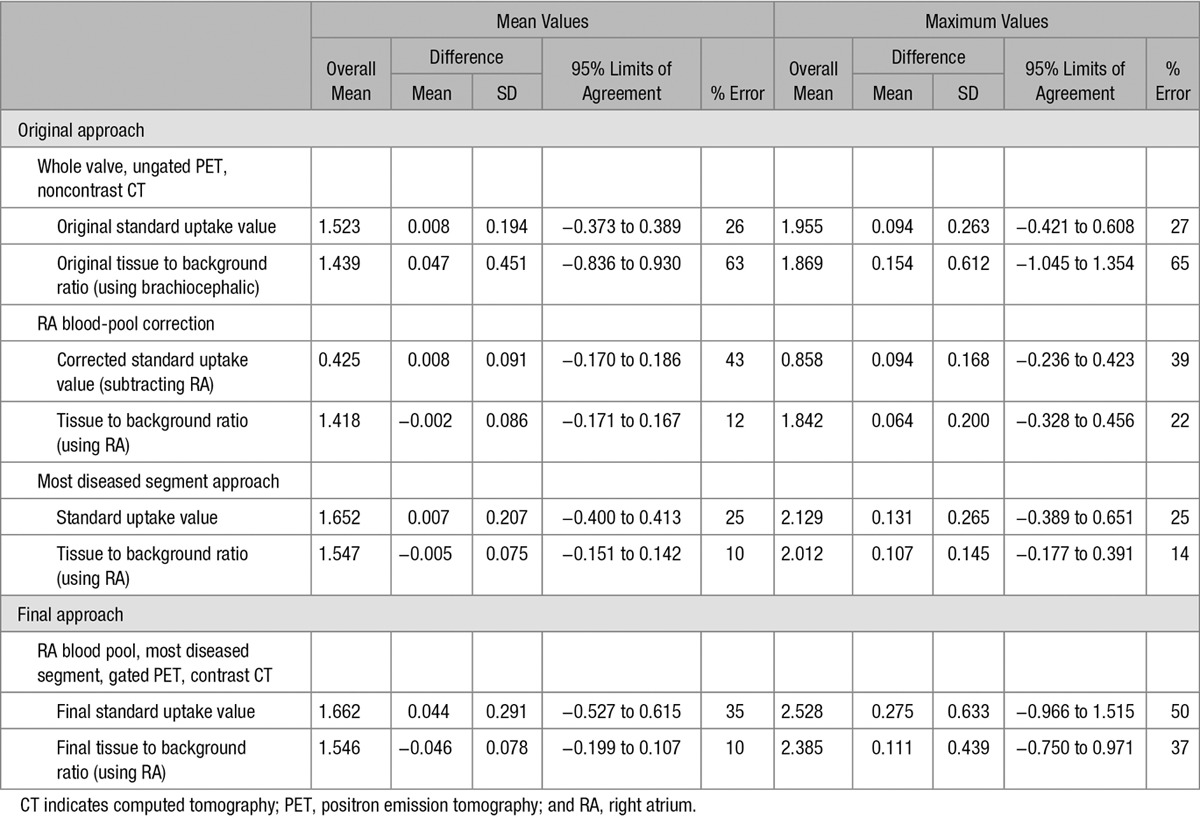
Bland–Altman Values and Percentage Errors for Each Stepwise Change to the Image Acquisition and Analysis Technique

**Figure 2.**
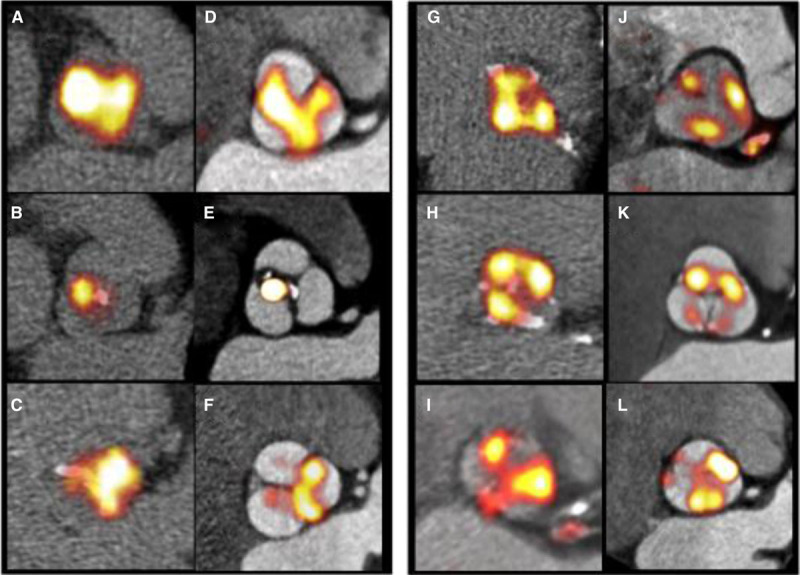
Improved localization of positron emission tomography (PET) signal within the aortic valve and its leaflets. Paired nongated, noncontrast PET/computed tomography (CT) scans (original approach **A**–**C** and **G**–**I**) and gated, contrast-enhanced PET/CT images (final approach **D**–**E** and **J**–**L**). Images demonstrate the typical distribution of the tracer uptake within the valve at sites of increased mechanical stress, that is, at the leaflet tips (**left**: **A**–**F**) and at the commissures (**right**: **G**–**L**).

On visual assessment, contrast CT imaging of the valve provided much clearer anatomic detail of the leaflets and valve structure compared with noncontrast CT (Figure 2). This made it technically easier to get into the true plane of the valve and allowed more accurate regions of interest to be drawn around its perimeter (Figures 1 and 2). Coregistration with ECG-gated PET data then allowed localization of 18F-fluoride uptake to individual leaflets and their different regions. This was previously impossible using noncontrast CT and nongated PET. Most commonly increased activity was observed across all 3 coronary cusps (n=10), it involved 2 cusps in 4 patients and was isolated to 1 cusp in just 1 patient. The noncoronary cusp was involved in all patients apart from that latter case. Activity was most frequently observed at the valve commissures: the point where the valve cusps meet the aortic ring (n=10) and at the tips where the leaflets coapt during diastole (n=8; Figure 2).

When examining intraobserver reproducibility for detecting the presence or absence of 18F-fluoride uptake on individual valve leaflets, this was very good for the right coronary cusp (κ=1.00), good for the noncoronary cusp (κ=0.63), and moderate for the left (κ=0.58) coronary cusps. The scan–rescan agreement was good for the right coronary cusp (κ=0.76), good for the noncoronary cusp (κ=0.63), and very good for the left coronary cusp (κ=0.81) coronary cusps (Tables 3 and 4).

**Table 3.**
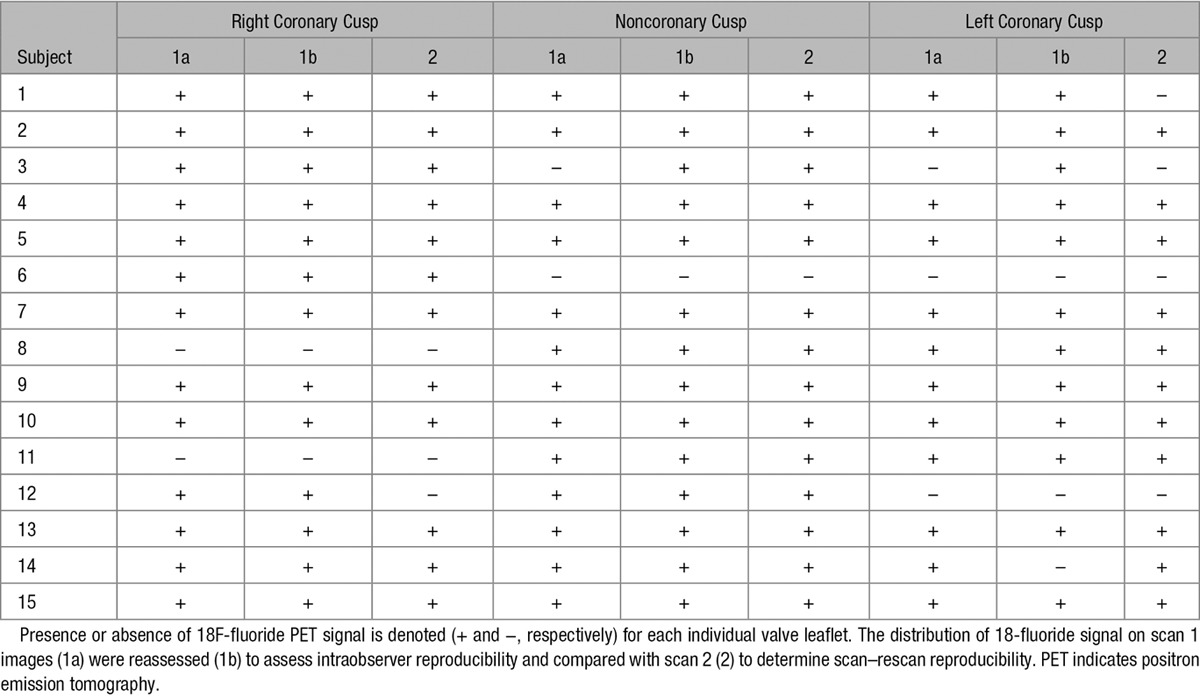
Scan–Rescan and Intraobserver Reproducibility for Presence or Absence of 18F-Fluoride Uptake

**Table 4.**
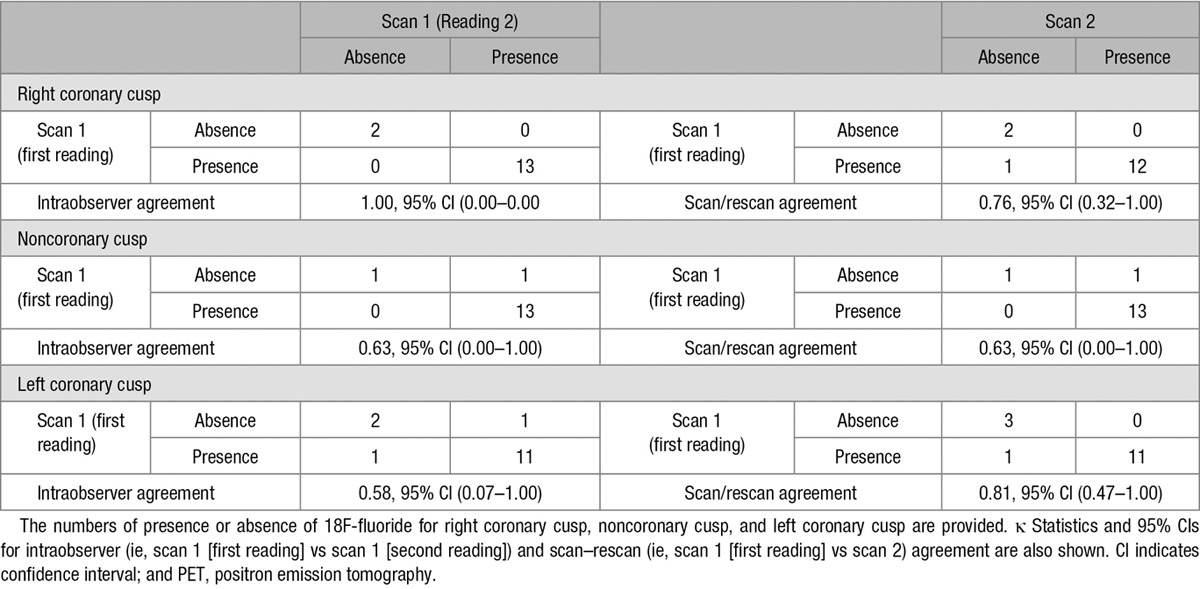
Kappa Statistics for Interobserver and Scan–Rescan Agreement for 18F-Fluoride PET Signal Distribution

### Effect of Altered Image Analysis on PET Reproducibility

Interobserver and intraobserver reproducibility was good using both the original and modified approaches as previously reported. Intraclass correlation coefficient values for intra- and interobserver reproducibility were 0.88 and 0.80, respectively (Table 5). However, the scan–rescan reproducibility of our original approach produced percentage errors of ±26% and ±27% for the mean and maximum SUV measurements, respectively (Table 2). Scan–rescan reproducibility for TBR measurements were disappointing with percentage errors of ±63% and ±65%.

**Table 5.**
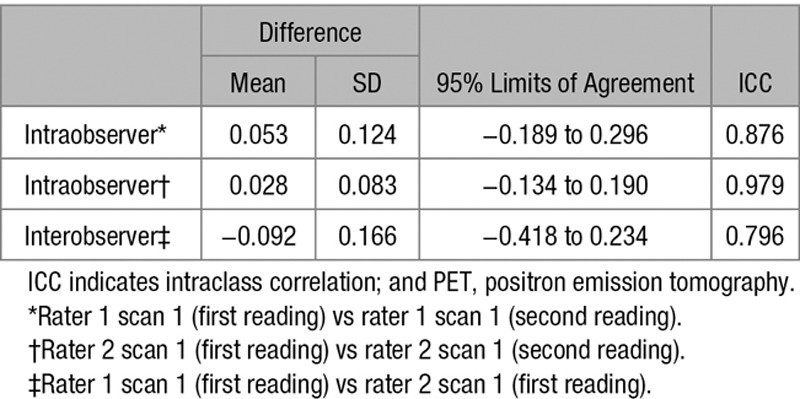
Intra-/Interobserver Variability of 18F-Fluoride PET Uptake (Expressed as a Continuous Variable)

### Blood-Pool Measurements

**Figure 3.**
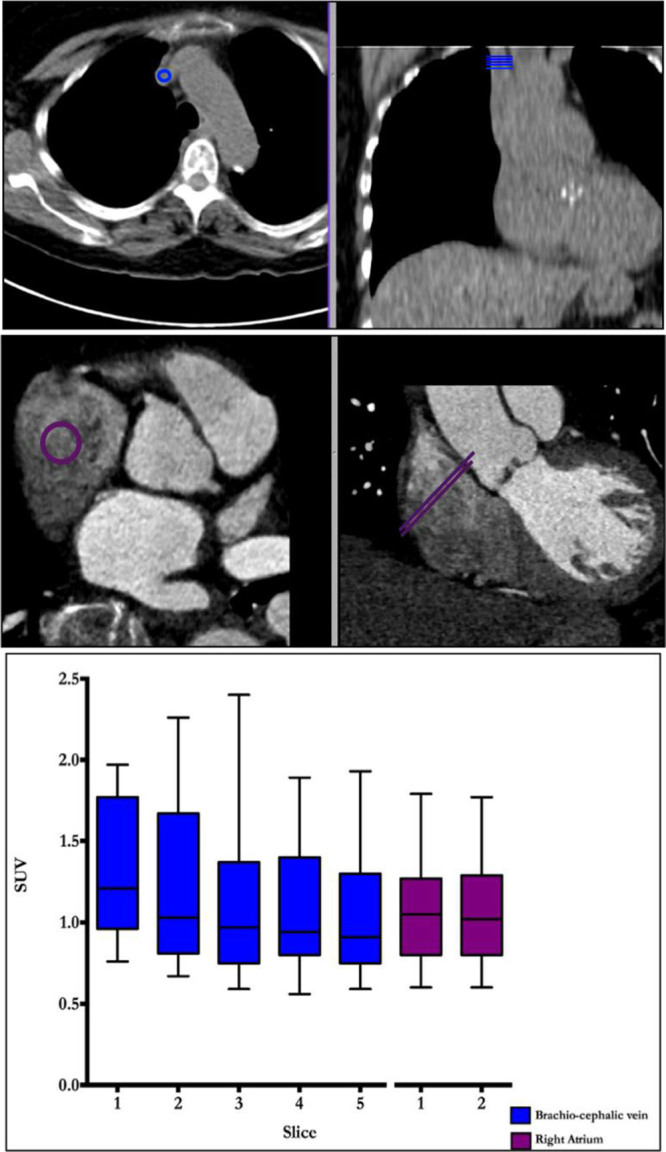
Measuring blood-pool activity in the brachiocephalic vein and the right atrium. Regions of interest for measuring blood-pool activity in the brachiocephalic vein (**top**) and right atrium (**bottom**) are shown in the en face of the valve (**left**) and coronal (**right**) planes. Note that the right atrium is a much larger structure allowing for larger regions of interest with less potential for partial volume artifact problems related to poor registration. Tukey plot demonstrates mean standard uptake values (SUV) for 5 contiguous slices from brachiocephalic (**blue**) and 2 from the right atrium (**purple**). Note the variation in brachiocephalic vein measurements between those taken most caudally vs those taken most cranially.

The percentage error of our original TBR values was double that of the SUV values, suggesting a problem with our blood-pool measurements. Interestingly, a stepwise and nonphysiological reduction in our original brachiocephalic vein measurements was observed on moving cranially up the axial slices away from the heart and into the lung. On average, a 20% difference in values was observed between the top and bottom slices, but this difference could be as high as 66%. By comparison, blood-pool sampling from the right atrium was easier to perform, allowed larger regions of interest to be drawn, and was consistent, demonstrating a <1% difference in measurements acquired on adjacent slices (Figure 3).

Sampling the blood pool in the right atrium led to a substantial improvement in the scan–rescan reproducibility of all our TBR measurements. Indeed, after implementing this approach, the reproducibility of our TBR values consistently outperformed those for SUV values with percentage errors of between ±12% and ±22% for mean and maximum values, respectively. In contrast, the approach of subtracting the blood-pool uptake from the tissue SUV to produce corrected aortic valve SUV measures did not greatly improve reproducibility resulting in percentage errors of ±43% and ±39% for mean and maximum measurements, respectively, despite similar limits of agreement (Table 2).

Considerable variation was observed in 18F-fluoride blood-pool PET activity across our population (blood-pool SUV 1.10±0.35) and even between different scans on the same patients. This is likely related to physiological variation in the distribution of the tracer.

### MDS Approach

The MDS technique improved the technical ease of image analysis, removing the difficulty in deciding on the upper and lower limits of the valve in the z-plane. This translated into further improvements in scan–rescan reproducibility for mean TBR values with the percentage error for TBR_MDSmean_ measurements reduced to ±10%. Similarly, maximum TBR values were optimized on addition of the MDS approach (percentage error TBR_MDSmax_ ±14%) as were the SUV measurements (percentage errors: SUV_MDSmean_ ±25%; SUV_MDSmax_ ±25%; Table 2).

### Final Approach: Addition of Contrast-CT and ECG-Gated PET

**Figure 4.**
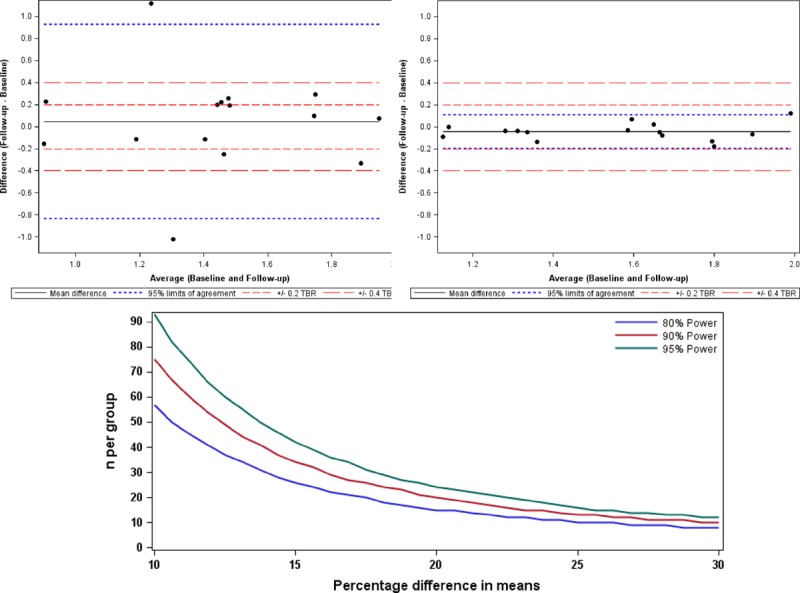
Scan–rescan reproducibility for 18F-fluoride positron emission tomography quantification in the aortic valve with consequent sample size estimates. Bland–Altman plots of scan–rescan reproducibility for tissue to background ratio (TBR)_MDSmean_ measurements using the original image analysis and acquisition methods (**left**) and then using final method (**right**). Percentage error for the final method is less than ±10%. Graph (**below**) shows the sample size estimates needed to detect differences in means that range from 10% to 30% of the initial scan point estimate. The plot illustrates the sample size required to detect differences in means ranging from 10% to 30% with figures shown for 80%, 90%, and 95% power. In all cases, this assumes that the common SD is 18.75%. MDS indicates most diseased segment.

The addition of contrast CT and ECG-gated PET data, while markedly improving image quality as described above, did not have a major effect on scan–rescan reproducibility. Reproducibility of our final approach, however, remained good with a percentage error of ±10% for TBR_MDSmean_ measurements (Figure 4). This, combined with its ability to localize PET uptake to individual leaflets, made the final approach our preferred strategy. TBR_MDSmean_ values using the final approach did not show any proportional bias with disease severity (Figure I in the Data Supplement) and were again superior to the equivalent SUV_MDSmean_ values (percentage error ±35%) and to measurements quantifying maximum valvular 18F-fluoride uptake, which were less reproducible after the addition of gated-PET and contrast-CT (percentage errors: SUV_MDSmax_ ±50% and TBR_MDSmax_ ±37%, respectively; Table 2).

## Discussion

In this study, we have systematically investigated the acquisition and analysis of 18F-fluoride PET imaging of the aortic valve. First, we have improved the spatial localization of tracer uptake using ECG-gated PET data and contrast CT imaging, so that activity can now be localized to individual leaflets and regions within those leaflets. This has demonstrated that calcification activity is most commonly observed at sites of maximal mechanical stress: in particular, in regions of leaflet coaptation and at the commissures. Second, we have improved the scan–rescan reproducibility by using blood-pool sampling of right atrium and the MDS methodology and ultimately demonstrated good agreement for repeat TBR_MDSmean_ measurements in the valve (percentage error ±10%). This has important implications for application to future clinical trials, indicating that 18F-fluoride might provide a useful imaging end point of drug efficacy.

In this study, we have modified our previous image acquisition protocol to include contrast-enhanced CT imaging of the aortic valve, thereby providing greater definition of the individual valve leaflets and their components. Moreover, we have included ECG-gated PET data to reduce the effects of cardiac motion and more accurately localize the pattern of activity on to the valve. The combined effect of these changes has been to improve the spatial localization of PET activity within the valve, which after accurate 3D coregistration, is now possible within specific regions of individual leaflets. This has demonstrated that 18F-fluoride activity predominantly localizes to sites of increased mechanical stress within the valve, supporting mechanical injury as a key driver to the disease process. For example, 18F-fluoride activity was observed at the edges of the valve leaflets exactly at the sites of leaflet impact during valve closure. Additionally, uptake was observed at the valve commissures where mechanical stress is concentrated before being transferred to the aortic wall.^12,13^ Although these findings need to be confirmed in larger studies with further refinement of thresholding techniques, they here provide key insight into the triggers to calcification activity in aortic stenosis and the importance of mechanical injury. Recent data have indicated that the relationship between the valve calcium burden and hemodynamic obstruction is not perfect.^14,15^ The ability of PET to accurately localize calcification activity may be useful in trying to understand whether calcium formation at different sites of the valve has different hemodynamic impacts.

We have modified our image analysis protocol, optimizing the scan–rescan reproducibility of 18F-fluoride imaging in the aortic valve using several different approaches. To date, it has been standard practice for 18F-fluorodeoxyglucose PET to measure the blood-pool SUV in the brachiocephalic vein.^16^ This has the benefit of avoiding contamination of myocardial 18F-fluorodeoxyglucose uptake that would overestimate the blood-pool activity if measured in the heart. However, this benefit does not exist for 18F-fluoride, which has no background myocardial uptake. We, therefore, measured blood-pool activity in both the right atrium and the brachiocephalic vein. Measurements in the right atrium are easily performed on the en face (short-axis) images of the valve and resulted in much more consistent blood-pool measurements. Moreover, this approach led to a dramatic improvement in the scan–rescan reproducibility of our TBR measurements such that they then outperformed equivalent SUV measures. We think that sampling the blood-pool activity in the right atrium improved reproducibility because these measurements are less susceptible to the partial volume effects of adjacent lung tissue and because any minor inaccuracies in coregistration with the PET signal will not have a great impact. Furthermore, it seems important to correct for variations in background blood-pool activity that can occur between scans perhaps because of minor changes in renal function, tracer dose, and pharmacokinetic distribution. Chen et al^9^ recently surmised that subtracting the blood pool from tissue SUV would improve accuracy. However, our study findings did not support this, and their approach produced lower TBR values, thereby increasing the percentage error of our repeat measurements.

Another major improvement in reproducibility was obtained using the MDS approach: measuring activity in the 2 hottest adjacent slices in the valve, rather than attempting to sample the entire valve. The major advantage of this technique is that it removes the considerable difficulty in deciding the limits and boundaries of the valve. Such uncertainty can lead to major differences in valve measurements because uptake is much lower at the extremes of the valve where the volume of tissue is small and inclusion of extraneous tissue will dilute down mean values.

In this article, although our stepwise changes to the protocol generally improved the reproducibility of mean measures of PET uptake, the effects on maximum measures were more variable. This finding is somewhat at odds with experience in oncology where the maximum values are often preferred. This may reflect the use of contrast-enhanced CT (not used in cancer imaging), which allowed accurate and reproducible regions of interest to be drawn around the perimeter of the valve, facilitating reproducible measurement of mean PET uptake. In addition, it may reflect ECG gating of the PET data, which discards 75% of the counts, potentially having a greater detrimental impact on maximum values (which rely on counts from only a few pixels and are therefore particularly susceptible to noise) than mean values. It is possible that advanced image analysis approaches that model and correct for cardiac motion without discarding any PET data will improve the reproducibility of TBR_MDSmax_ measurements as has recently been described for coronary 18F-fluoride activity.^17^

This is the first study to assess scan–rescan reproducibility for 18F-fluoride uptake in the aortic valve. For a technique to be clinically applicable, clinicians and clinical researchers need the reassurance that a given methodology is robust and reproducible. We have demonstrated this here. However, we acknowledge that scan–rescan reproducibility does not necessarily translate to accuracy and sensitivity. The value of 18F-fluoride as an imaging marker of calcification activity will ultimately be determined by its ability to predict disease progression and to detect changes in calcification activity in response to novel therapies. These aspects are both currently being studied within the SALTIRE 2 clinical trial. We have already shown that the TBR_MDSmean_ can predict disease progression and clinical events in patients with aortic stenosis.^18^ We can now report TBR_MDSmean_ measurements, made using our optimized image acquisition and analysis protocols, can quantify valvular 18F-fluoride activity with good reproducibility and a 10% error. This translates directly into the requirement for low patient numbers for studies investigating the effects of interventions on 18F-fluoride PET uptake (as a marker of calcification activity), because any true effect will not be swamped by noise within the measuring technique. Indeed, based on our reproducibility data, we have provided estimates of the sample sizes required for different effect sizes (Figure 4). For example, 57 patients would be required in each group to detect a 10% difference in mean 18F-fluoride activity based on 80% power and an α error probability of 0.05. However, although these estimates provide a framework for minimum sample sizes, they should be interpreted with a degree of caution, because they assume a perfect agreement between changes in the TBR_MDSmean_ signal and underlying changes in valve calcification activity.

### Limitations

We acknowledge the small sample size in this study; however, it is similar to that used in previous studies examining the reproducibility of vascular PET^19^ and, in part, reflects attempts to minimize the radiation exposure associated with repeat PET/CT imaging. Moreover, although previous studies have indicated that 18F-fluoride uptake correlates with histological markers of calcification activity and accurately predicts the progression in the CT calcium score, we currently lack data to show that 18F-fluoride is modifiable with drug therapy. Largely, this is because no drug has yet demonstrated an ability to reduce disease activity in aortic stenosis, and we lack reliable animal models of this condition.

In conclusion, we have optimized 18F-fluoride PET-CT imaging in the aortic valve. Excellent localization of the PET signal within the aortic valve is now possible, with uptake observed in regions of maximal mechanical stress. Moreover, quantification of valvular 18F-fluoride uptake is now possible with good scan–rescan reproducibility. 18F-Fluoride PET-CT holds major promise as a method to better understand calcification activity in aortic stenosis and as a surrogate end point in clinical trials assessing the efficacy of potential therapeutic interventions.

## Acknowledgments

We acknowledge the support of staff at the Edinburgh Heart Centre at the Royal Infirmary of Edinburgh and the radiography and radiochemistry staff of the Clinical Research Imaging Centre. We also acknowledge the Edinburgh Clinical Trials Unit and the Trial Steering Committee for the SALTIRE 2 clinical trial.

## Sources of Funding

The study was funded by the British Heart Foundation (FS/14/78/31020). Drs Pawade, Cartlidge, Jenkins, Dweck, and Newby are supported by the British Heart Foundation (SS/CH/09/002/26360, FS/13/77/30488, SS/CH/09/002/2636, FS/14/78/31020, and CH/09/002). Dr Newby is the recipient of a Wellcome Trust Senior Investigator Award (WT103782AIA). Dr Dweck is the recipient of the Sir Jules Thorn Award for Biomedical Research 2015. Dr Adamson is supported by New Zealand Overseas Training and Research Fellowship (1607) and Edinburgh and Lothians Health Foundation (50–534). The Wellcome Trust Clinical Research Facility and the Clinical Research Imaging Centre are supported by NHS Research Scotland (NRS) through NHS Lothian. Dr Rudd is partly supported by the NIHR Cambridge Biomedical Research Centre, the British Heart Foundation, and the Wellcome Trust.

## Disclosures

None.

## Supplementary Material

## CLINICAL PERSPECTIVE

18F-Fluoride positron emission tomography (PET) is being increasingly used as a research tool to study calcification activity in the vasculature. It binds preferentially to regions of newly developing microcalcification beyond the resolution of computed tomography (CT) and, in aortic stenosis, correlates closely with calcification activity on histology (alkaline phosphatase staining). Moreover, clinical 18F-fluoride PET-CT in aortic stenosis offers accurate prediction of disease progression and adverse cardiovascular events. 18F-Fluoride PET-CT, therefore, has major potential to improve our understanding of the role of calcification in aortic stenosis and also as a surrogate end point in studies of novel therapies for this condition. This article adds to current literature demonstrating that optimized 18F-fluoride PET-CT in aortic stenosis can localize calcification activity to individual leaflets and regions of the valve associated with increased mechanical stress. Moreover, it shows that the scan–rescan reproducibility of aortic valve PET measurements is good, with a percentage error of ±10%. These data strongly support the use of 18F-fluoride PET-CT as a tool to study the mechanisms underlying aortic stenosis and also as an end point in novel therapies for this common condition.
