# Supplementary material for: Optimization and Reproducibility of Aortic Valve 18F-Fluoride Positron Emission Tomography in Patients With Aortic Stenosis
Source: Circ Cardiovasc Imaging. 2016 Oct 18;9(10):e005131. doi: 10.1161/CIRCIMAGING.116.005131 (PMC5068186; doi:10.1161/CIRCIMAGING.116.005131)

SUPPLEMENTAL MATERIAL

Table 1. Full list of inclusion and exclusion criteria

**Inclusion Criteria**	Age >50 years
	Peak aortic jet velocity of >2.5 m/s on Doppler echocardiography
	Grade 2-4 calcification of the aortic valve on echocardiography
**Exclusion Criteria**	Women of childbearing potential who have experienced menarche, are premenopausal,
	Women who have not been sterilized or who are currently pregnant.
	Women who are breastfeeding
	Renal failure (estimated glomerular filtration rate of <30 mL/min)
	Inability to undergo scanning
	Allergy or contraindication to iodinated contrast
	Inability or unwilling to give informed consent
	Likelihood of non0compliance to treatment allocation or study protocol.
	Anticipated or planned aortic valve surgery in the next 6 months,
	Life expectancy less than 2 years,
	Treatment for osteoporosis with bisphosphonates or denosumab.

	Known allergy or intolerance to alendronate or denosumab, or any of their excipients<br>Abnormalities of the oesophagus or conditions, which delay oesophageal/gastric emptying.<br>Inability to sit or stand for at least 30 minutes.<br>Hypocalcaemia<br>Regular calcium supplementation<br>Dental extraction within 6 months<br>Long term corticosteroid use.<br>History of osteonecrosis of the jaw<br>Poor dental hygiene<br>Major or untreated cancers

Figure 1. Linear regression analysis of scan-rescan reproducibility versus calcium burden. The mean difference between 18F-Fluoride TBRMDSmean measures for the 15 scan pairs is plotted against the log-transformed Computed Tomography calcium score (log_{10} Agatston Units). No proportional bias is observed.

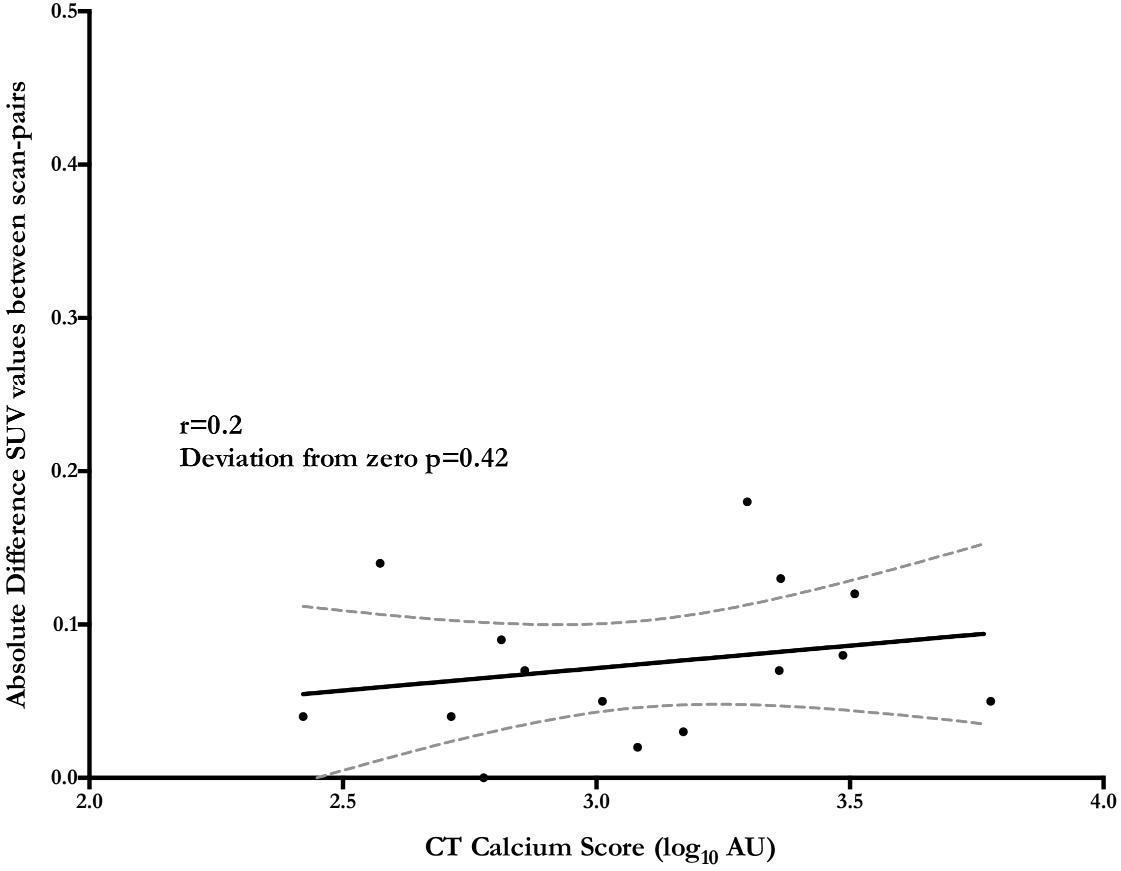

Supplement: Supplementary file 1 [file hci-9-e005131-s001.pdf]
